# A new species of *Zeraikia* Gil-Santana & Costa with taxonomic notes on *Zeraikia novafriburguensis* Gil-Santana & Costa (Hemiptera, Reduviidae, Peiratinae)

**DOI:** 10.3897/zookeys.716.20843

**Published:** 2017-11-29

**Authors:** Hélcio R. Gil-Santana

**Affiliations:** 1 Laboratório de Diptera, Instituto Oswaldo Cruz, Av. Brasil, 4365, 21040-360, Rio de Janeiro, RJ, Brazil

**Keywords:** corsairs, Heteroptera, male genitalia, Neotropics, taxonomy

## Abstract

*Zeraikia zeraikae* **sp. n.** is described from the state of Rio de Janeiro, Brazil, based on one male and one female specimens. Some taxonomic notes on *Zeraikia* Gil-Santana & Costa, 2003 and *Zeraikia novafriburguensis* Gil-Santana & Costa, 2003 are provided. Detailed descriptions and several figures of the male genitalia of both species are furnished. A key for the species of *Zeraikia* is presented.

## Introduction

There are 11 genera of Peiratinae in the Neotropics (Maldonado 1990, Cai and Taylor 2006, Melo 2012). A summary of the taxonomic bibliography of this group in the Neotropical region and an updated key to the genera recorded there were furnished by Gil-Santana et al. (2015).

*Zeraikia* Gil-Santana & Costa, 2003 and *Zeraikia novafriburguensis* Gil-Santana & Costa, 2003 were described based on a single male that was collected in Nova Friburgo, a city located in a mountainous region of the state of Rio de Janeiro, Brazil (Gil-Santana and Costa 2003).

Following publication of this species, another male of *Z. novafriburguensis* was located in the Entomological Collection of the National Museum of the Federal University of Rio de Janeiro (MNRJ) and a female of the species was collected in the same locality in which the holotype was found. Through this opportunity, some additional details about this species are furnished, including a redescription of the male genitalia. Photographs of the holotype are also provided for the first time.

*Zeraikia zeraikae* sp. n. is described from Nova Friburgo, Brazil, based on one male and one female specimens.

A revised diagnosis and description of *Zeraikia* are presented.

## Material and methods

The specimens described here are or will be deposited in the Entomological Collection of the “Museu Nacional da Universidade Federal do Rio de Janeiro”, Rio de Janeiro, Brazil (MNRJ). When citing the text on the labels of a pinned specimen, a slash (/) separates the lines and a double slash (//) different labels. All measurements are in millimeters (mm).

**Figures 1–3.**
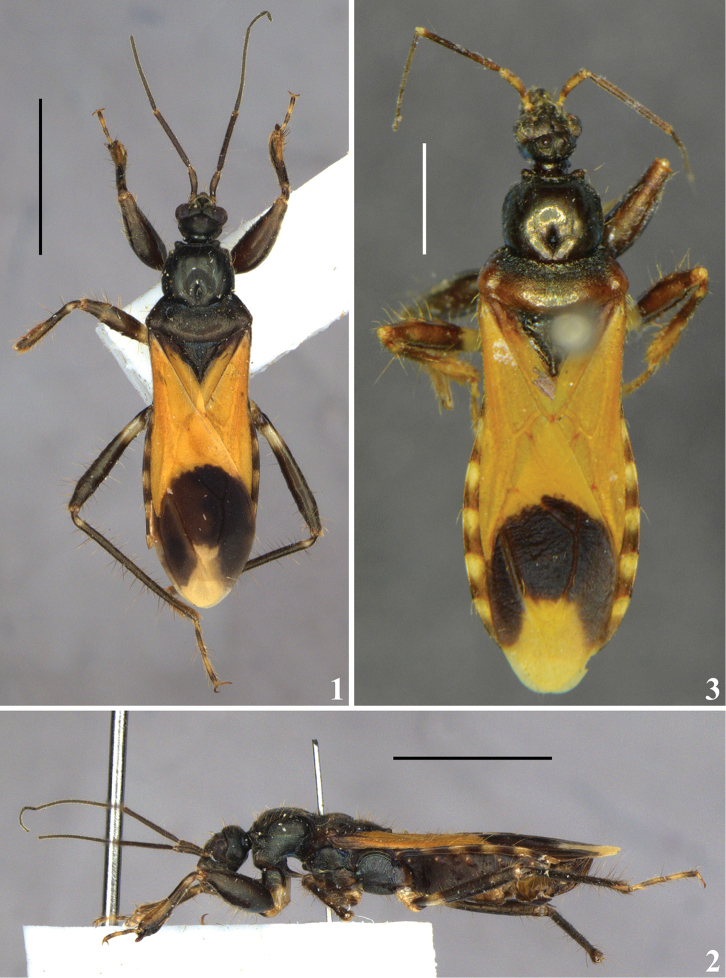
*Zeraikia novafriburguensis*, male. **1–2** holotype (courtesy of the team of the digitization project of the Entomological Collection of MNRJ and Luiz A. A. Costa) **1** dorsal view **2** lateral view **3** additional specimen, dorsal view. Scale bars: 5.0 mm (**1–2**); 2.0 mm (**3**).

The photos of the male holotype of *Z. novafriburguensis* (Figs 1–2) were kindly provided by the team of the digitization project of the Entomological collection of MNRJ (“Projeto Informatização da Coleção Entomológica do Museu Nacional/UFRJ, SIBBR/CNPq proc. 405588/2015–1”). These photos were taken with a Leica DFC450 C camera attached to a Leica M205 C stereomicroscope. Multiple focal planes were merged using the auto-montage software Leica Application Suite version 4.8.0.

**Figures 4–7.**
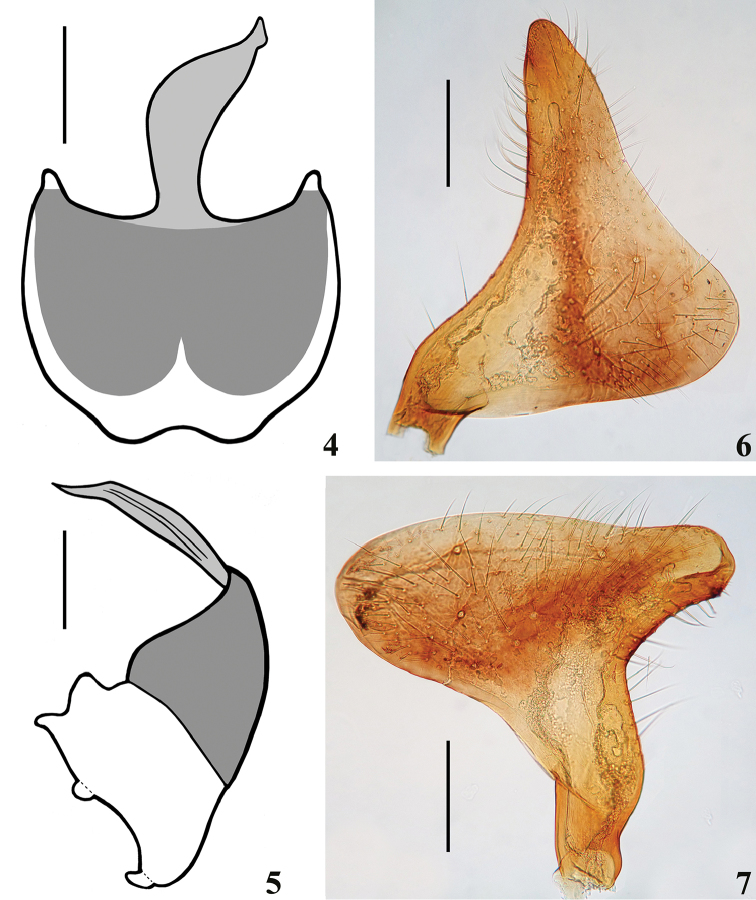
*Zeraikia novafriburguensis*, male genitalia. **4–5** pygophore without parameres **4** ventral view **5** lateral view **6** left paramere **7** right paramere. Scale bars: 0.5 mm (**4–5**); 0.3 mm (**6–7**).

All remaining figures were produced by the author. The fixed adults, microscopic preparations, and genitalia were photographed using digital cameras (Nikon D5200 with a Nikon Macro Lens 105 mm and Sony DSC-W830). Drawings were made using a camera lucida. For clarity, the vestiture (setation) was completely or almost completely omitted in the ink drawings of Figs 4–5, 21–25, 41–42. Images were edited using Adobe Photoshop 7.0.

**Figures 8–11.**
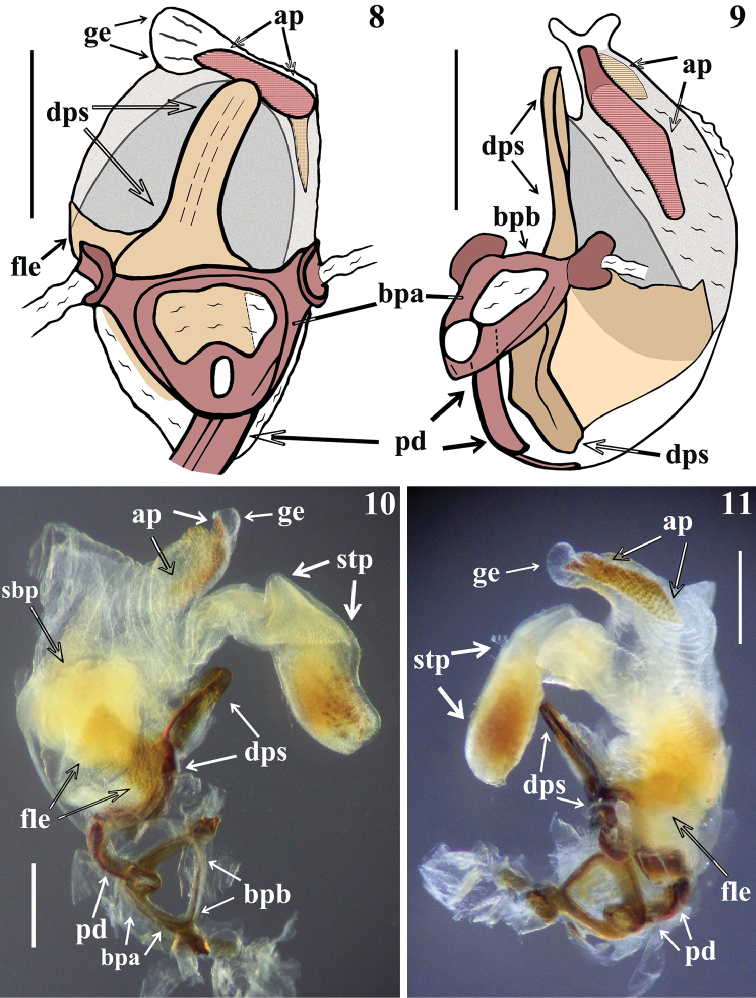
*Zeraikia novafriburguensis*, male genitalia. **8–9** phallus not inflated **8** dorsal view **9** latero-dorsal view **10–11** phallus with endosoma inflated, lateral views. (**ap** apical process of endosoma; **bpa** basal plate arm; **bpb** basal plate bridge; **dps** dorsal phallothecal sclerite; **fle** flat lateral expansion; **ge** globose expansion of endosoma; **pd** pedicel; **sbp** subbasal process of endosoma; **stp** subapical tubular projection of endosoma wall). Scale bars: 0.5 mm.

**Figures 12–16.**
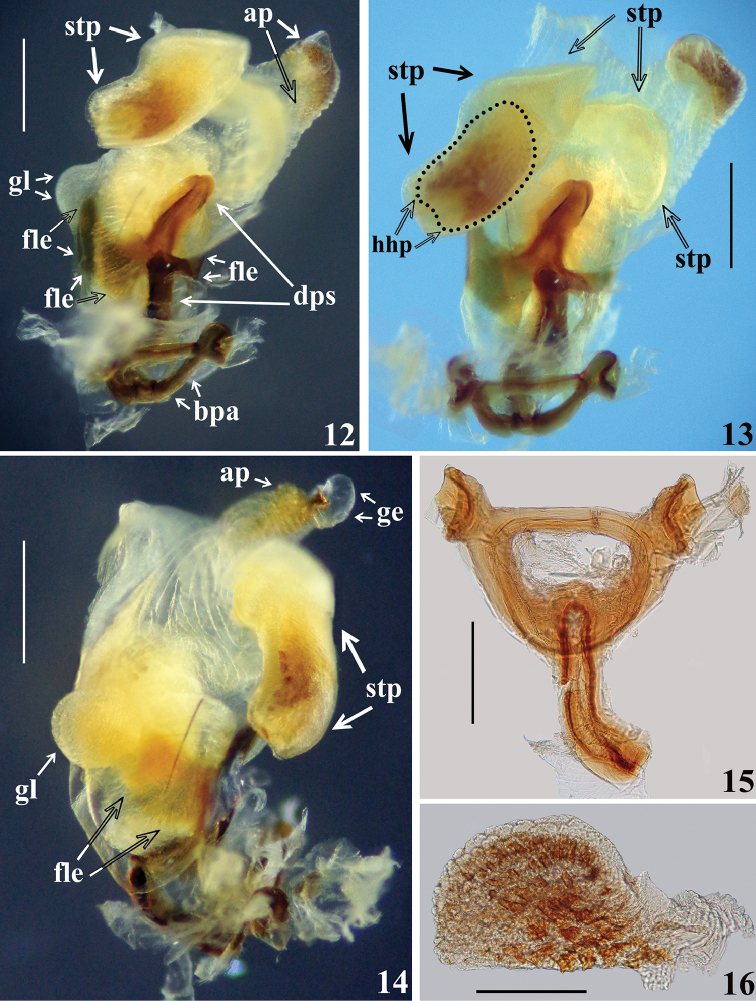
*Zeraikia novafriburguensis*, male genitalia. **12–14** phallus with endosoma inflated **12–13** dorsal view **14** lateral view **15** articulatory apparatus and pedicel **16** half-hemispherical process. (**ap** apical process of endosoma; **bpa** basal plate arm; **dps** dorsal phallothecal sclerite; **fle** flat lateral expansion; **ge** globose expansion of endosoma; **gl** small lateral globose lobe of endosoma wall; **hhp** half-hemispherical process; **stp** subapical tubular projection of endosoma wall). Scale bars: 0.5 mm (**12–14**); 0.3 mm (**15–16**).

Observations were made using a Zeiss Stemi stereoscope microscope and a Leica CME compound microscope. Measurements were made using a micrometer eyepiece. The total length of the head was measured excluding the neck, for better uniformity of this measurement. Dissections of the male genitalia were made by first removing the pygophore from the abdomen with a pair of forceps and then clearing it in 20% NaOH solution for 24 hours. Following this procedure, the phallus of each species was firstly recorded without inflation (Figs 8–9, 31–32). The dissected structures were studied and photographed in glycerol. In the case of the holotype of *Z. zeraikae* sp. n., there was inadvertent breakage of the basal plate bridge of the articulatory apparatus and loss of one arm of it (Figs 32–33). The endosoma was then everted by carefully pulling on the endosoma wall, using a pair of fine forceps, especially on the visible portion of the long tubular process, in order to release it completely from the surrounding tissues (Figs 10–14, 32, 34).

General morphological terminology mainly follows Schuh and Slater (1995). Currently, there is a lack of consensus about the terminology to be applied to female and male genitalia in Reduviidae (see Forero and Weirauch 2012). Therefore, in order to maintain uniformity with previous works (e.g. Cai and Taylor 2006), the terminology of the genitalia structures follows Lent and Wygodzinsky (1979). However, the “vesica”, as recognized by the latter authors, has been considered to be absent in reduviids. The assumed equivalent structure in reduviids is a somewhat sclerotized appendage of the phallosoma of the endosoma (Forero and Weirauch 2012) but not the homologous vesica of other heteropterans, such as Pentatomomorpha (Rédei and Tsai 2011). Thus, this term is not used here.

In general, the features already recorded in the description of *Zeraikia* are not repeated in the descriptions provided for its species.

## Taxonomy

### Subfamily Peiratinae

#### Zeraikia

Taxon classificationAnimaliaHemipteraReduviidae

Gil-Santana & Costa, 2003

Zeraikia Gil-Santana & Costa, 2003: 4 [key; description], 7 [discussion]; Forero 2004: 155 [diagnosis], 155–156 [comments], 187 [key]; Cai and Taylor 2006: 51 [citation], 54 [Key]; Gil-Santana et al. 2015: 327 [key].

##### Type species.

*Zeraikia novafriburguensis* Gil-Santana & Costa, 2003: 4–7, by monotypy.

##### Diagnosis.

Head somewhat longer than fore lobe of pronotum; transverse sulcus distinct, shallowly impressed. Postocular region in dorsal view converging to neck from a point considerably posterior to eyes. Meso and metapleural sutures curved. Prosternal process ending short of apices of fore coxae. Space between mid coxae and hind coxae slightly smaller and wider than coxal diameter, respectively. Fore and mid tibia with spongy fossa. Spongy fossa of fore tibia occupying somewhat less than half length of the tibia, ventrally; spongy fossa of mid tibia about half size in comparison with that of fore tibiae, occupying approximately distal fourth of mid tibia, ventrally.

##### Description.

Small sized (maximum length 10.7–12.8). *Structure*: integument mostly shiny. **Head**: integument mostly finely granulose and rugulose; on postocular region and lateral tubercles of neck there are larger granules; shorter than pronotum; somewhat longer than fore lobe of pronotum; anteocular portion longer than postocular and elongated. Transverse sulcus distinct, shallowly impressed with a small anterior median depression. Eyes of medium size, somewhat more than half as wide as interocular space in dorsal view, reaching near and far from outline of head dorsally and ventrally, respectively; in lateral view, at level of midportion of labial segment III (second visible). Distance between ocelli approximately or somewhat more than twice diameter of ocellus. Area immediately posteroventral to eyes, somewhat prominent. Antennae inserted near eyes, elongated; segment I shortest and thickest, enlarged toward apex; remaining segments longer and progressively thinner. Labium robust, segment III (second visible) longest; segment IV thinnest, tapering; integument smooth on segments III–IV. Postocular region in dorsal view converging to neck from a point considerably posterior to eyes. Neck with a conspicuous pair of lateral tubercles, integument generally smooth and shiny. **Thorax**: integument mostly finely or coarsely granulose and rugulose; pronotal collar moderately developed, with prominent lateral lobes. Fore lobe of pronotum longer than hind lobe, lateral margins rounded in dorsal view; hind lobe enlarged toward posterior margin; lateral margins of fore and hind lobe carinate; humeral angles rounded and posterior margin convex; transverse furrow well-marked, curved, subparallel to posterior margin; fore lobe strongly longitudinally depressed in middle of posterior third, with shallow medial sulcus at anterior two-thirds and similar lateral sulci; integument finely granulose on the sulci and mostly shiny and smooth among sulci. Scutellum triangular; apical margin rounded. Prothoracic acetabula broad. Meso and metapleural sutures curved. Prosternal process elongated, tapering, ending short of apices of fore coxae, with stridulitrum occupying its midline, ventrally. Meso and metasternum finely punctuated. Mesosternum with a midlongitudinal shallow crest. Fore coxae thickened, somewhat thinner toward apices, elongated, implanted near each other; space between them smaller than width of each coxa and occupied by the prosternal process. Mid and hind coxae subglobular, progressively more widely separated from each other; space between mid coxae and hind coxae slightly smaller and wider than coxal diameter, respectively. Fore femora thickest, hind femora longest, mid femora somewhat thicker than hind femora. Fore and mid tibiae slightly shorter than respective femora and bearing spongy fossa. Fore tibiae conspicuously enlarged and somewhat curved upwards at approximately apical half; spongy fossa large, occupying somewhat less than half length of the tibia, ventrally, and surpassing its apex approximately to level of basal portion of second tarsomere. Mid tibiae somewhat enlarged at apical third to apical fourth; spongy fossa about half size in comparison with that of fore tibiae, occupying approximately distal fourth of the tibia, ventrally, and surpassing its apex approximately to the level of middle portion of first tarsomere. Hind tibiae slightly longer than femora, almost entirely straight, sometimes somewhat thickened or curved subapically. Hemelytra generally dull, not surpassing tip of abdomen in females but extending somewhat beyond it in males; on extreme base of dorsal surface, laterally, a small, somewhat elevated, translucent, rugulose area present. **Abdomen** suboval, larger in females; dorsal connexival segments also more prominent in females. Integument very finely rugulose and granulose. Sternite II (first visible) with dull and rugulose integument, granulose on median portion, sometimes also finely granulose on lateral portions. Sternites III–VII with shiny integument, variably rugulose; their median portion generally smoother, mainly in females, in which the segments are more enlarged; on sternite III a shallow median carina, sometimes incomplete, not evident on posterior half of the segment. In males, only posterior margin of sternite VIII visible, in which there is a median posterior elongated subtriangular prolongation with the apex rounded; the remaining segment, i.e., the non-exposed part, less pigmented and less sclerotized; basal margin curved backwards on midportion ventrally. Integument somewhat rugulose on exposed portion of genital segments.

*Male genitalia*: asymmetrical. Exposed portion of pygophore subquadrate to subrectangular in ventral view; its non-exposed portion almost unpigmented and less sclerotized; basal margin curved backwards on midportion ventrally; median process large, elongated, strongly curved in ventral view, somewhat narrower at basal and apical portions; tip of apex blunt in ventral view and acute in lateral view. Parameres subtriangular, margins rounded; left paramere slightly longer than right paramere, with its apical portion more slender; a subapical process (**sp**) present on inner surface of both parameres, small on left paramere and larger on right paramere; just below this process, at one side, a longitudinal crest (**lc**) present on inner surface, somewhat shorter and more prominent on right paramere. Phallus suboval in shape when not inflated. Articulatory apparatus with moderately short basal plate arms (**bpa**); basal arms and basal plate bridge (**bpb**) forming a subtriangular set; basal plate bridge somewhat narrower than basal plate arms; pedicel (**pd**) moderately elongated, curved in lateral view. Dorsal phallothecal sclerite (**dps**) asymmetrical, twice curved in lateral and dorsal views, elongated; at its approximately median third there is a pair of asymmetrical rounded flat lateral expansions (**fle**); somewhat depressed at median portion, longitudinally, on dorsal surface of approximately distal half; apex rounded in dorsal view. Endosoma with three main processes: 1 – a globose to elongate subbasal larger process (**sbp**) formed by diffuse thickening; two processes formed by several to numerous variably sclerotized elements; 2 – an apical, elongated curved process (**ap**), formed by numerous sclerotized spined elements, in which the tip is surrounded by a globose expansion of endosoma (**ge**); and 3 – another process, which varies in shape, located basally or apically inside the subapical tubular projection of endosoma (**stp**). Endosoma wall longitudinally and transversely finely striated; finely rugose in some portions, especially on apical part; sometimes forming a globose small lateral lobe (**gl**); with a very long, conspicuous, subapical tubular projection (**stp**) which is apparent only after careful detachment and expansion of it apart from the surrounding tissues; in a resting position, most part of it remains embedded in phallosoma. Length of this tubular projection, when fully stretched, approximately as long as or somewhat longer than anteroposterior length of phallus.

*Female genitalia*: simple. Tergites IX and X obliquely directed backwards, clearly separated by a thin line; in posterior view, subtrapezoidal and subrectangular in shape, respectively.

*Vestiture*: integument generally covered by numerous, short, adpressed, pale to silvery setae, sometimes forming a pubescence; long to very long, darkened, stiff, straight or variably curved setae and stiff, darkened, straight or variably curved moderately short setae. **Head** covered with pale to silvery pubescence which is somewhat rarer ventrally and in postocular region, and also with scattered long oblique darkened stiff setae. Neck glabrous, except on lateral tubercles which are covered by pubescence. Eyes glabrous. Antenna: segment I less setose; pubescence and sparse, stiff, obliquely curved, short setae more numerous toward apex, with a few, longer, stiff, somewhat curved to straight, darkened setae, one of them, implanted on approximately median portion of dorsal surface, is conspicuously thicker than the others. Segments II and III covered by dense pubescence formed by numerous thin, oblique, short pale setae, and scattered, longer, stiff, oblique, darkened setae. On segment II, some trichobothria present, which are variable in length, some of them very long; a conspicuous, subbasal, strong, stiff, straight, very long, darkened, dorsal seta and a subapical, dorsal, somewhat long, stiff, somewhat curved darkened seta. On segment IV, the setae forming the dense pubescence are even thinner and more adpressed, while the stiff oblique setae are somewhat longer, thinner and more numerous along the segment. Labium less setose, segments III and IV with some scattered stiff long straight setae only. **Thorax** with short, pale pubescence and variable long, stiff, straight or variably curved setae. Lateral lobes of pronotal collar with a single conspicuous, stiff, long, darkened seta. Integument glabrous on shiny and smooth areas among sulci of fore lobe of pronotum. Femora and tibiae with scattered, conspicuous, long to very long, straight, stiff, darkened setae, with variable areas also covered with pale pubescence. Pubescence on tibiae generally denser, mainly on ventral and dorsal surfaces of mid and hind tibiae; towards apical portion of the segment, tufts formed with very numerous, stiff, short, oblique, yellowish to golden-yellowish setae on ventral surface of fore tibiae, and dorsal and ventral surfaces of mid and hind tibiae. Tarsi densely covered with stiff, pale, yellowish to golden-yellowish, oblique to curved setae of variable lengths, somewhat shorter and more numerous on ventral surfaces of the segments. Hemelytra moderately setose on lateral portions of base and costal area of corium, sparsely on other portions, in which most if not all the setae are implanted on veins, with glabrous areas among them; membrane glabrous. **Abdomen**: each dorsal connexival segment with a single conspicuous long, stiff, darkened, somewhat curved to straight setae implanted just above posterolateral angle. Pubescence formed by very short and adpressed setae on connexivum and lateral portions of sternites; the latter with variable number of scattered long stiff darkened setae. In males, posterior margin of segment VII with some to several, very long, conspicuous, somewhat curved, stiff, darkened setae. **Genitalia** covered by short thin setae and longer stiff scattered setae on exposed portions. Parameres glabrous on basal portion; covered by numerous, moderately long, thin setae and a few, conspicuously longer, larger setae scattered on the exposed (outer) surface; inner surface mostly glabrous with a few rows of moderately long, thin setae basally to the inner crest and on the latter, in which the setae are more numerous and longer.

#### Zeraikia novafriburguensis

Taxon classificationAnimaliaHemipteraReduviidae

Gil-Santana & Costa, 2003

Figures 1–3 , 4–7 , 8–11 , 12–16 , 17–18

Zeraikia novafriburguensis Gil-Santana & Costa, 2003: 4, 7 [description], 5–6 [figures 1–10]; Forero 2004: 155–156 [citation]; Gil-Santana et al. 2015: 326 [citation].

##### Material examined.

*Zeraikia novafriburguensis*. **Type material**. **BRAZIL**, Rio de Janeiro, Nova Friburgo Municipality, Cascatinha neighborhood (22°20'S, 42°33'W, ca. 1000 m a.s.l.), **Holotype** (male): Reduviidae / Peiratinae // Holotipo [red label] // *Zeraikia* / *novafriburguensis*. / Hélcio Gil-Santana / &. Luiz A. A. Costa, 2003 // 28-IX-2001 – Casca- / tinha – N. Fribur- / go – RJ – BRASIL // [QR CODE] / MNRJ-ENT3-142 (MNRJ).

##### Additional specimens.

**BRAZIL**, Santa Catarina, Corupá Municipality, x. 1944, 01 male; Nova Friburgo, Cascatinha neighborhood (22°20'S, 42°33'W, ca. 1000 m a.s.l.), 19.xii.2003, 01 female, (MNRJ).

##### Diagnosis.

General coloration blackish to brownish black, sometimes with subtle bluish luster, mainly on sternites. General coloration of corium, clavus and basal portion of membrane of hemelytra orange to reddish orange. Labial segments III and IV, coxae, trochanters, tibiae and tarsi distinctly or extensively marked with pale to yellowish pale markings. Pale markings of connexivum large, occupying approximately basal half of each segment. Several long stiff dark setae on ventral surface of femora, mainly on fore and mid femora, implanted in small rounded tubercles.

##### Addition to the original description.

Male. Figures 1–17. Measurements (holotype /additional male): total length: to tip of hemelytra: 10.7 / 11.0; to tip of abdomen: 10.2 / 10.4; abdomen maximum width: 3.0 / 3.2.

*Coloration*: general coloration blackish to brownish black, sometimes with subtle bluish luster, mainly on sternites (Figs 1–3). Head with the following pale to pale yellowish parts: base and a submedian distal ring, somewhat variable in extent on antennal segment I; apex of antennal segment II; most of basal two-thirds of labial segment III (second visible), and apices of the labial segments III and IV. Posterior margin of hind lobe of pronotum somewhat paler in the additional male (Fig. 3). Apex of prosternal process paler. Coxae pale yellowish in approximately apical two-thirds. Trochanters pale yellow with apices darkened. Mid and hind femora with moderately large, subbasal, pale yellow rings. Fore femora with a small, almost imperceptible, paler, subapical spot, dorsally. Mid femora with a narrow, interrupted or incomplete, subdistal pale ring. Tibiae with a narrow, pale, subbasal ring. Fore and mid tibiae with apical half paler, of variable extent around the segment. Tarsi generally pale to pale yellow, with segments II and III darkened at base and apex. Hemelytra (Figs 1, 3): most of corium and clavus and basal portion of membrane orange to reddish orange; corium and clavus darkened to blackish in basal portion; most of membrane blackish with a median apical large pale yellow spot, somewhat enlarged towards apex, and somewhat larger in the additional male. Connexivum with approximately basal half of segments III–VII pale to pale yellow (Figs 1, 3).

*Structure and vestiture*: generally as in generic description. Tip of scutellum horizontal, directed backwards. Several long stiff dark setae on ventral surface of femora, mainly on fore and mid femora, implanted in small rounded tubercles. Hemelytra covered with numerous adpressed setae on costal area and numerous and longer stiff setae on veins of corium, surpassing abdomen by about half a millimeter.

**Figures 17–18.**
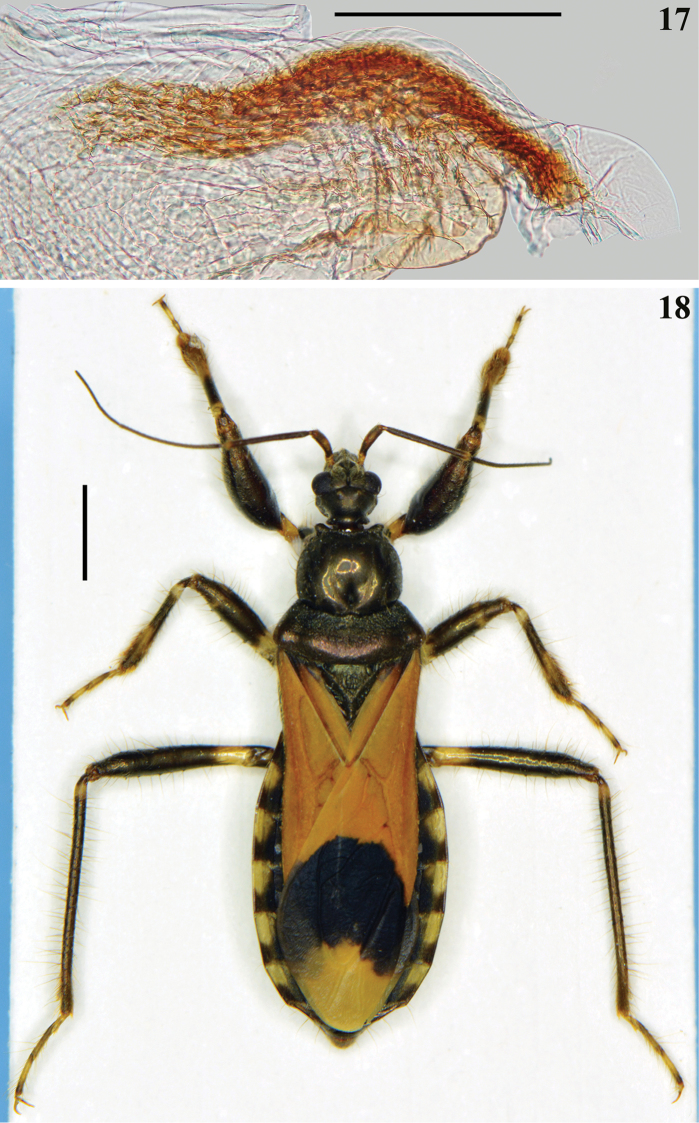
*Zeraikia novafriburguensis*. **17** male genitalia, apical process of endosoma (**ap**) **18** female, dorsal view. Scale bars: 0.3 mm (**17**); 2.0 mm (**18**).

*Male genitalia* (in addition to generic characteristics described above) (Figs 4–17): both subapical processes of parameres spiniform. Dorsal phallothecal sclerite (**dps**): very curved in lateral view at basal third (Figs 9–11); flat lateral expansions of the mid third (**fle**) prolonged laterally to approximately half the distance between dorsal and ventral surfaces (Figs 10–11, 14); left (in dorsal view) flat lateral expansion somewhat more than half the width of opposite flat lateral expansion (Figs 10–14); apical third of dorsal phallothecal sclerite almost straight in lateral view (Figs 9, 11). Diameter of subapical tubular projection of endosoma wall (**stp**) roughly uniform, with a half-hemispherical process (**hhp**) inside its apical portion (Figs 10–14), which is covered by several small sclerotized components, some of which are acutely spined (Fig. 16). Endosoma wall with a small rounded globose lobe laterally (**gl**), located just ventrally to apex of larger flat lateral expansion of dorsal phallothecal sclerite (Figs 12, 14).

Female. Figure 18. Measurements: total length to tip of abdomen: 12.5; abdomen maximum width: 4.0. Similar to male. Hemelytra not surpassing abdomen, ending shortly after posterior margin of dorsal portion of segment VIII; pale yellow distal spot of the membrane somewhat larger at basal half, subrounded. Abdomen larger; dorsal connexival segments somewhat more evident.

##### Distribution.

Brazil, in states of Rio de Janeiro and Santa Catarina.

#### Zeraikia zeraikae sp. n.

Taxon classificationAnimaliaORDOFAMILIA

http://zoobank.org/A15DABA5-F9EA-4FDA-BE26-B6A48E61AAA2

Figures 19 , 20–25 , 26–30 , 31–34 , 35–38 , 39–42

##### Type material.

**BRAZIL**, Rio de Janeiro, Nova Friburgo Municipality (22°17'S, 42°29'W, ca. 1049 m a.s.l.), 05.xi.1997, **Holotype** (male); iii.1996, **Paratype** (female).

##### Diagnosis.

General coloration black with bluish luster. General color of corium, clavus and basal half of membrane of hemelytra dull blackish. Labium, coxae, trochanters, tibiae and tarsi mostly or completely darkened, without conspicuous pale markings. Pale markings of connexivum relatively small, occupying approximately basolateral third and fifth of segments III–VI and VII, respectively. Long stiff dark setae on ventral surface of femora relatively smaller and not implanted in tubercles.

##### Description.

Male. Figures 19–38. Measurements are given in Table 1.

**Figure 19.**
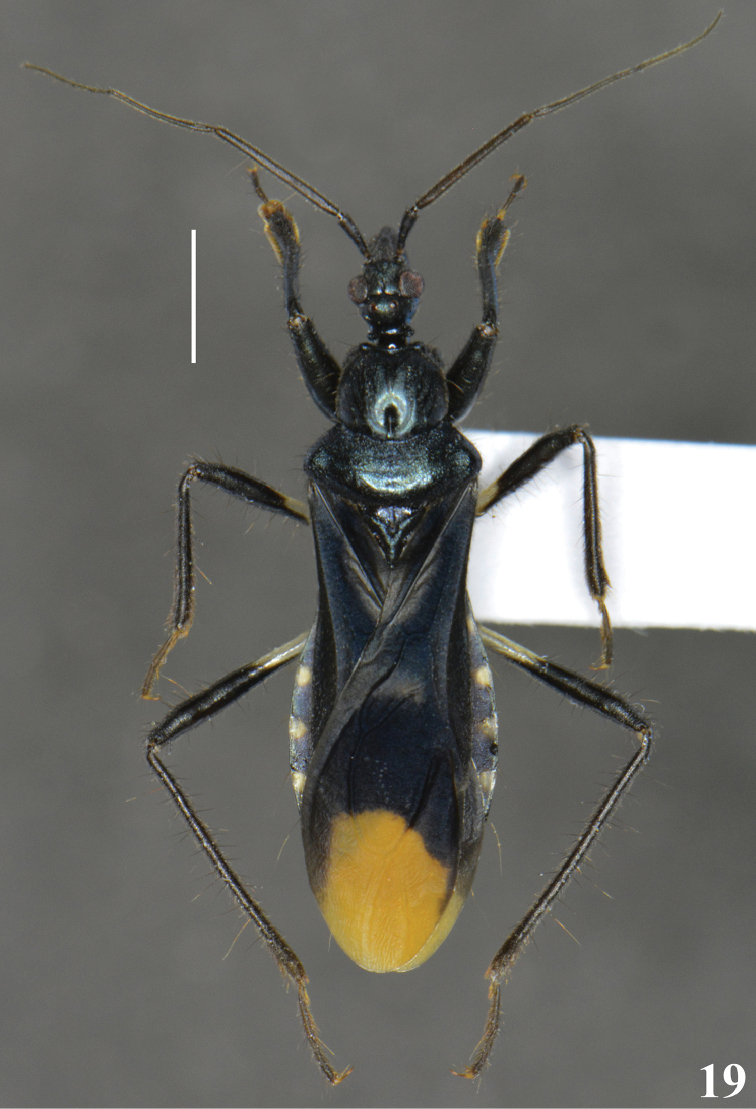
*Zeraikia zeraikae* sp. n., male holotype, dorsal view. Scale bar: 2.0 mm.

**Figures 20–25.**
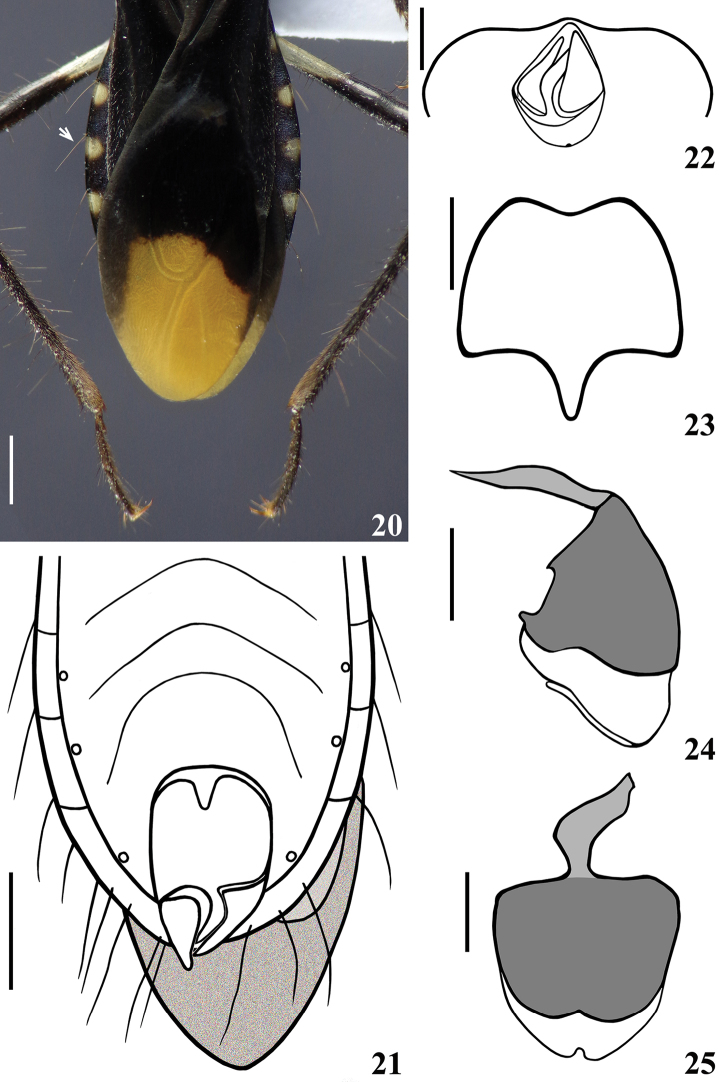
*Zeraikia zeraikae* sp. n., male holotype. **20** distal portion of hemelytra and connexivum, dorsal view (the arrow points to a single conspicuous long, stiff, darkened seta implanted just above posterolateral angle of a connexival segment) **21** distal portion of abdomen, ventral view **22** apical portion of abdomen, posterior view (hemelytra omitted) **23** sternite VIII, ventral view **24–25** pygophore without parameres **24** lateral view **25** ventral view. Scale bars: 1.0 mm (**20–21**); 0.5 mm (**22–25**).

*Coloration*: general coloration black with bluish luster, mostly very shiny, except on hemelytra, which are completely dull (Fig. 19). Extreme base of antennal segment I pale whitish. Apex of labial segment III (second visible) paler; apical half of labial segment IV reddish brown. Extreme base of trochanters somewhat paler; extreme apex of mid and hind trochanters pale. Mid and hind femora with a subbasal whitish marking that occupies the dorsal and lateral surfaces, but does not form a ring because it does not reach the ventral surface; larger on hind femora. Fore tarsomere I paler. Hemelytra: apical portion of clavus and adjacent portion of corium with a faintly smoky grayish marking just below level of apex of scutellum; an arcuate marking with similar coloration present just above mid portion of hemelytra, which crosses median third of upper closed cell, running as far as inner (posterior) margin; the latter somewhat paler to faintly smoky grayish from this arcuate marking to level of approximately mid portion of membrane (Figs 19–20). Membrane with a large subrounded yellowish orange spot at approximately distal half (Figs 19–20). Pale whitish spots on basolateral third and fifth of connexival segments III–VI and VII, respectively (Figs 19–20).

*Structure and vestiture*: generally as in generic description. Tip of scutellum directed obliquely upwards. Long stiff dark setae on ventral surface of femora relatively smaller and not implanted in tubercles. Hemelytra with thinner and less numerous setae in costal area and on veins of corium, surpassing abdomen by somewhat more than one millimeter.

**Figures 26–30.**
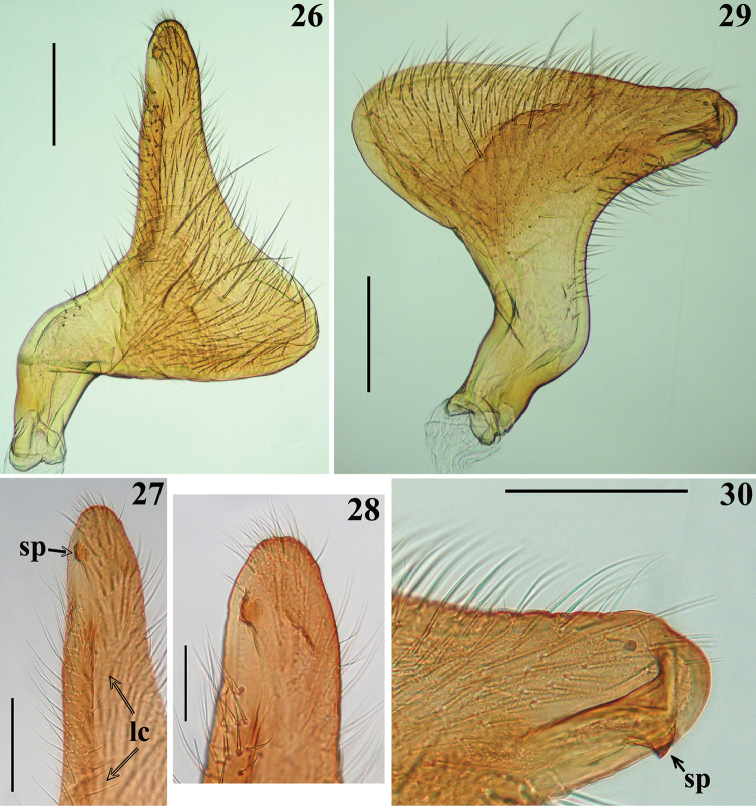
*Zeraikia zeraikae* sp. n., male genitalia, parameres. **26–28** left paramere **27–28** apical portion **29–30** right paramere **30** apical portion. (**sp** subapical process; **lc** longitudinal crest). Scale bars: 0.3 mm (**26, 29**); 0.2 mm (**27, 30**); 0.1 mm (**28**).

**Figures 31–34.**
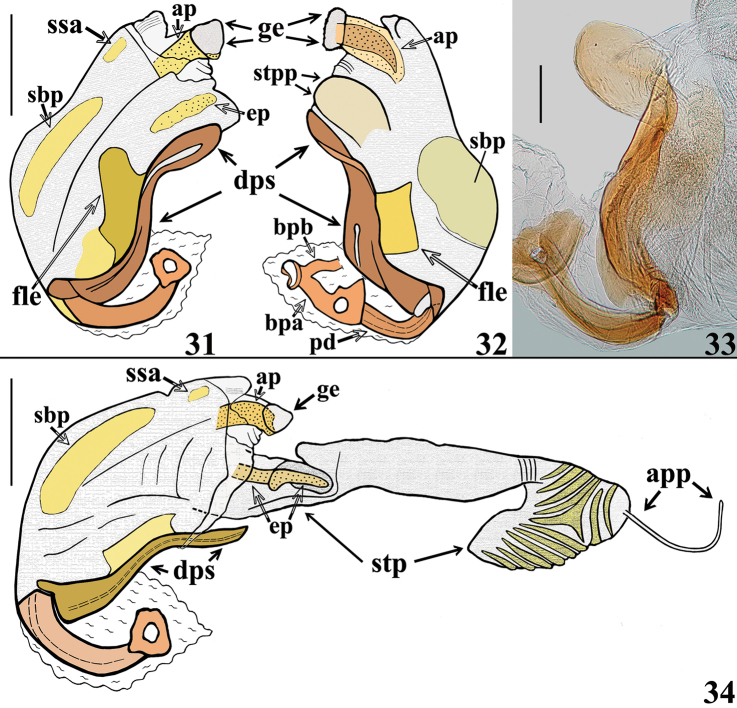
*Zeraikia zeraikae* sp. n., male genitalia, lateral views. **31–32** phallus not inflated **33** pedicel and dorsal phallotecal sclerite **34** phallus with endosoma inflated. (**ap** apical process of endosoma; **app** appendix; **bpa** basal plate arm; **bpb** basal plate bridge; **dps** dorsal phallothecal sclerite; **ep** elongated process inside basal portion of subapical projection of endosoma wall; **fle** flat lateral expansion; **ge** globose expansion of endosoma; **pd** pedicel; **sbp** subbasal process of endosoma; **ssa** small subapical process of endosoma; **stp** subapical tubular projection of endosoma wall; **stpp** visible portion of subapical tubular projection not extended). Scale bars: 0.5 mm (**31–32, 34**); 0.2 mm (**33**).

**Figures 35–38.**
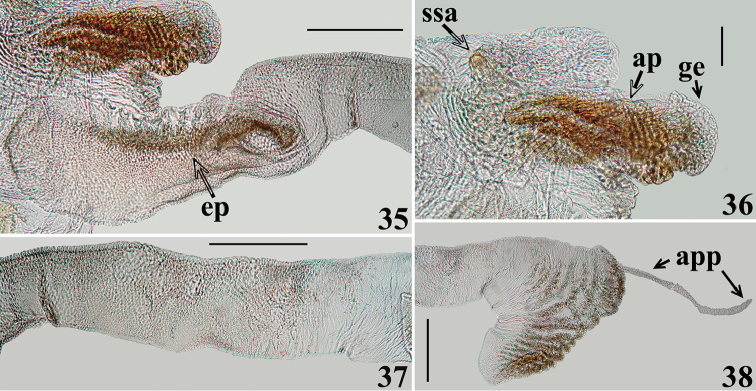
*Zeraikia zeraikae* sp. n., male genitalia, lateral views. **35** apical process and basal portion of subapical tubular projection of endosoma wall. (**ep** elongated process inside basal portion of subapical tubular projection of endosoma wall) **36** small subapical and apical processes of endosoma. (**ap** apical process; **ge** globose expansion of endosoma **ssa** small subapical process) **37–38** endosoma wall projection portions **37** midportion **38** apical portion. (**app** appendix). Scale bars: 0.3 mm (**35, 37–38**); 0.1 mm (**36**).

*Male genitalia* (in addition to generic characteristics described above) (Figs 21–38): subapical process (**sp**) of right paramere spiniform (Fig. 30); blunt on left paramere (Figs 27–28). Dorsal phallothecal sclerite (**dps**) (Figs 31–34): less curved in lateral view at basal third; flat lateral expansions (**fle**) of mid third prolonged laterally approximately only to a third of distance between dorsal and ventral surfaces; faintly sclerotized; left (in dorsal view) flat lateral expansion (**fle**) approximately a third larger than right flat lateral expansion; apical third of dorsal phallothecal sclerite slightly curved in lateral view. Subapical tubular projection of endosoma wall (**stp**) conspicuously enlarged at apical portion, which is subtriangular in shape, with several low rugose crests and a very thin elongated appendix (**app**) at its tip, which is rounded (Figs 34, 37–38); inside its basal portion, an elongated, thin, slightly curved process (**ep**) formed by numerous, small, subtriangular to spined, somewhat sclerotized elements (Figs 34–35). An additional very small, rounded, somewhat sclerotized, subapical process (**ssa**) present just ventral to basal portion of the elongated curved apical process (**ap**) (Figs 31, 34, 36).

**Figures 39–42.**
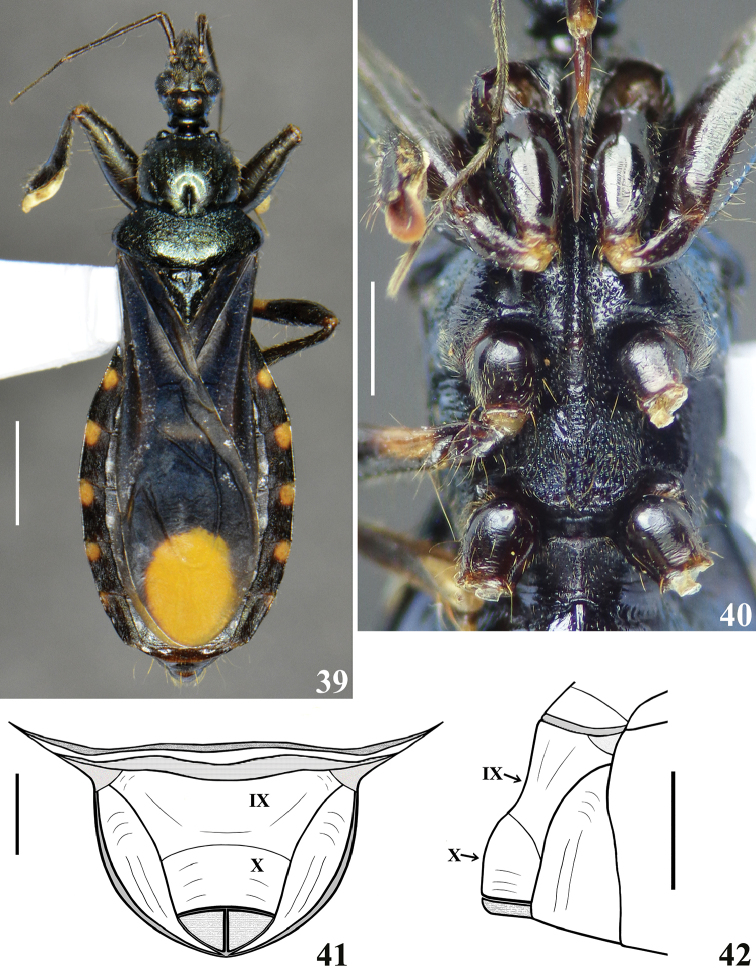
*Zeraikia zeraikae* sp. n., female paratype. **39** dorsal view **40** thorax, ventral view **41–42** female genitalia **41** posterior view **42** lateral view. (**IX** tergite IX; **X** tergite X). Scale bars: 2.0 mm (**39**); 1.0 mm (**40**); 0.5 mm (**41–42**).

Female. Figures 39–42. Similar to male in general. Measurements presented in Table 1. Pale markings on femora and connexivum yellow to yellowish orange (Fig. 39). Hemelytra not surpassing abdomen, ending shortly before posterior margin of tergite VII; large yellowish orange spot at distal half of membrane smaller and more rounded in shape (Fig. 39). Tip of scutellum straight, directed upwards. Abdomen larger; dorsal connexival segments more evident (Fig. 39).

**Table 1.** Measurements of *Zeraikia zeraikae* sp. n.

**Measurements**	**Holotype male**	**Paratype female**
Body length to tip of hemelytra	11.9	-
Body length to tip of abdomen	10.8	12.8
Head length (excluding neck)	2.0	2.0
Anteocular portion length	1.0	1.0
Postocular portion length	0.4	0.4
Head width across eyes	1.2	1.3
Interocular distance	0.5	0.6
Transverse width of eye	0.35	0.4
Antennal segment I length	0.7	0.7
Antennal segment II length	2.4	2.0
Antennal segment III length	1.8	1.5
Antennal segment IV length	2.0	1.8
Labial segment II length	0.6	0.6
Labial segment III length	1.0	1.0
Labial segment IV length	0.7	0.7
Ocellar tubercle width	0.5	0.5
Fore lobe of pronotum length	1.5	1.6
Fore lobe of pronotum max. width	1.8	2.0
Hind lobe of pronotum length	1.0	1.0
Hind lobe of pronotum max. width	2.8	3.0
Scutellum length	1.0	1.0
Scutellum maximum width	1.2	1.2
Fore coxa length	1.5	1.6
Fore femur length	2.7	2.8
Fore tibia length	2.3	2.5
Spongy fossa of fore tibia length	1.4	1.5
Spongy fossa of fore tibia max. width	0.5	0.5
Fore tarsus length	0.9	absent
Mid femur length	2.4	2.7
Mid tibia length	2.6	2.7
Spongy fossa of mid tibia length	0.7	0.7
Spongy fossa of mid tibia max. width	0.2	0.3
Mid tarsus length	1.2	1.2
Hind femur length	3.4	3.6
Hind tibia length	4.4	4.5
Hind tarsus length	1.6	1.7
Abdomen length	5.2	6.5
Abdomen maximum width	3.4	4.3

##### Etymology.

The new species is named in honor of my beloved wife, Soraya Orichio Zeraik, who has always stimulated and helped me in my entomological studies.

##### Distribution.

Brazil, in state of Rio de Janeiro.

## Discussion

The overall similarities in structure and vestiture between *Z. novafriburguensis* and *Z. zeraikae* sp. n. justify their placement in the same genus. The characteristics of *Z. zeraikae* sp. n. also corroborate placement of this species in *Zeraikia* in accordance with the definition of this genus by Gil-Santana and Costa (2003) and the diagnostic criteria of the keys furnished by Cai and Taylor (2006) and Gil-Santana et al. (2015).

On the other hand, *Z. novafriburguensis* and *Z. zeraikae* sp. n. can be unequivocally separated from each other based on several characteristics that are recorded in their respective diagnoses presented above. These and other additional differences, mentioned in their descriptions, are clear-cut enough to undoubtedly consider them to be distinct species.

In the male genitalia, the most striking differences were recorded between the characteristics of the long subapical tubular projection of the endosoma wall (**stp**) (Figs 10–14, 34–35, 37–38) and its associated process in each species (Figs 16, 35). In this regard, it is noteworthy that it was observed here that detachment and expansion of this long tubular projection of the endosoma wall from the surrounding tissues requires special attention. Because it does not seem to expand spontaneously, it might otherwise remain unnoticed and unrecorded. This structure not only showed features distinctive to the species studied here but also only after its release was it possible to study the rest of the endosoma and its processes in their entirety.

The general shape of the exposed portion of the eighth sternite, the pygophore, including its median process and parameres in these species of *Zeraikia* (Figs 4–7, 21–26, 29) are similar to several species belonging to other Neotropical genera of Peiratinae, such as *Eidmannia* Taeuber, 1934, *Lentireduvius* Cai & Taylor, 2006 and *Rasahus* Amyot & Serville, 1843, as described in previous studies by Coscarón (1986), Cai and Taylor (2006) and Coscarón (1983), respectively. The shape and structure of the articulatory apparatus in *Zeraikia* (Figs 8–11, 13, 15, 32–33) is similar to that of several other Peiratinae e.g. *Rasahus*, *Lentireduvius*, *Sirthenea* Spinola, 1837 (Coscarón 1983, Cai and Taylor 2006, Willemse 1985) or even several other Reduviidae, e.g. Triatominae (Lent and Wygodzinsky 1979).

A similar long tubular expansion of the endosoma wall, like that of *Z. novafriburguensis* (with a roughly uniform diameter) (Figs 10–14), was recorded for *Lentireduvius brasiliensis* Cai & Taylor, 2006 by Cai and Taylor (2006), although they considered it to be part of the “vesica”, a term that is not used here. The dorsal phallothecal sclerite of *L. brasiliensis* seemed similar to that of *Z. zeraikae* sp. n., and a lobe similar to the globose small lateral lobe of the endosoma wall in *Z. novafriburguensis* (**gl**) (Figs 12, 14) was also recorded in *L. brasiliensis* (Cai and Taylor 2006).

On the other hand, because of the lack of more complete data about other structures of the endosome, including its processes, in previous works, further comparisons will require additional studies. In this regard, it is worth mentioning that, in almost all studies on Neotropical Peiratinae (see Gil-Santana et al. 2015 for an updated bibliography), the endosoma was not really or fully everted, which might have prevented a more detailed description of these structures.

### Key for the species of *Zeraikia*

**Table:** 

1	General coloration blackish to brownish black, sometimes with subtle bluish luster, mainly on sternites (Figs 1–3, 18). General coloration of corium, clavus and basal portion of membrane of hemelytra orange to reddish orange (Figs 1, 3, 18). Pale markings of connexivum large, occupying approximately the basal half of each segment (Figs 1–3, 18)	***novafriburguensis* Gil-Santana & Costa**
–	General coloration black with bluish luster (Figs 19, 39). General color of corium, clavus and basal half of membrane of hemelytra dull blackish (Figs 19–20, 39). Pale markings of connexivum relatively small, occupying approximately the basolateral third and fifth of segments III–VI and VII, respectively (Figs 19–20, 39)	***zeraikae* sp. n.**

## Supplementary Material

XML Treatment for Zeraikia

XML Treatment for Zeraikia novafriburguensis

XML Treatment for Zeraikia zeraikae
